# Effects of Exposure to Physical Factors on Homeopathic Preparations as Determined by Ultraviolet Light Spectroscopy

**DOI:** 10.1100/tsw.2010.15

**Published:** 2010-01-08

**Authors:** Barbara Marschollek, Mathias Nelle, Martin Wolf, Stephan Baumgartner, Peter Heusser, Ursula Wolf

**Affiliations:** ^1^ Institute of Complementary Medicine KIKOM, University of Bern, Switzerland; ^2^ Division of Neonatology, University Hospital Bern, Switzerland; ^3^ Hiscia Institute, Arlesheim, Switzerland

**Keywords:** UV spectroscopy, optical properties, physical properties, chemical properties, homeopathy, anthroposophic medicine, exposure

## Abstract

Clinical trials have reported statistically significant and clinically relevant effects of homeopathic preparations. We applied ultraviolet (UV) spectroscopy to investigate the physical properties of homeopathic preparations and to contribute to an understanding of the not-yet-identified mode of action. In previous investigations, homeopathic preparations had significantly lower UV light transmissions than controls. The aim of this study was to explore the possible effects of external factors (UV light and temperature) on the homeopathic preparations. Homeopathic centesimal (c) dilutions, 1c to 30c, of copper sulfate (CuSO_4_), decimal dilutions of sulfur (S_8_), 1x to 30x, and controls (succussed potentization medium) were prepared, randomized, and blinded. UV transmission was measured at six different time points after preparation (from 4 to 256 days). In addition, one series of samples was exposed to UV light of a sterilization lamp for 12 h, one was incubated at 37°C for 24 h, and one was heated to 90°C for 15 min. UV light transmission values from 190 or 220 nm to 340 nm were measured several times and averaged. After each exposure, UV transmission of the homeopathic preparations of CuSO_4_ was significantly reduced compared to the controls, particularly after heating to 37°C. Overall, the nonexposed CuSO_4_ preparations did not show significantly lower UV transmission compared to controls; however, the pooled subgroup of measurements at days 26, 33, and 110 yielded significant differences. UV light transmission for S_8_ preparations did not show any differences compared to controls. Our conclusion is that exposure to external factors, incubation at 37°C in particular, increases the difference in light transmission of homeopathic CuSO4 preparations compared to controls.

### INTRODUCTION

Homeopathic preparations are applied in homeopathy and anthroposophic medicine, two types of complementary medical disciplines relatively widespread in Europe. Homeopathy is based on the simile principle. This means that symptoms in a person are treated with potentized substances that, if ingested undiluted, lead to similar symptoms in a healthy person[1]. Homeopathic preparations or potencies are produced by diluting and rhythmically succussing a mother tincture. This procedure is known as the "potentization process". At high dilution levels, the probability of the presence of molecules of the original substance in the preparation is almost zero. Therefore, mode of action cannot be explained by the presence of a chemical ingredient. Nevertheless, clinical effectiveness of homeopathic preparations was reported in several clinical trials[2,3,4]. Several hypotheses about the mode of action have been presented[5,6,7,8,9,10,11,12,13,14], but none of these have been proven so far. The investigation and determination of physical properties may provide evidence to clarify the mode of action of homeopathic preparations.

In previous studies, homeopathic preparations had different UV absorption characteristics from controls[15,16,17,18,19,20]. In our previous study[21] and our own pilot studies (unpublished data), we found a higher UV light absorption for highly diluted homeopathic copper sulfate ($CuSO_4$) preparations than for controls. This may be interpreted as a less structured or more dynamic diluent, or a higher intermolecular energy of the homeopathic preparations. Therefore, our research seeks to further investigate UV light absorption and to analyze whether external physical forces influence homeopathic preparations. This may provide more information on the physical properties and stability of homeopathic preparations under various conditions, which is of particular interest since in modern life, homeopathic preparations are often exposed to physical forces such as high temperature and pressure (e.g., autoclavation), artificial magnetism, ionizing radiation (e.g., scanners at airports, train stations), or nonionizing radiation (e.g., mobile communication devices). These influences may have effects on the stability and quality of homeopathic preparations. Thus, the aim of this study was to investigate differences between homeopathic preparations of $CuSO_4$ and sulfur ($S_8$) and corresponding controls, their dependence on storage time (from 4 to 256 days), and possible effects generated by exposing homeopathic preparations to UV light and to two different temperatures, 37 and 90ºC.

### MATERIALS AND METHODS

#### Chemicals and Water

Homeopathic preparations were produced from copper sulfate ($CuSO_4 \cdot 5H_2O$) (Weleda AG, Arlesheim, Switzerland) and sublimed sulfur $S_8$ (Phytomed AG, Hasle/Rueegsau, Switzerland). Homeopathic preparations and controls were prepared with autoclaved, distilled, and deionized water with an electrical resistance of 18 MΩ (Hiscia Institute, Arlesheim, Switzerland). For cleaning, 18 MΩ distilled sterile Aqua B. Braun® water was used.

#### Vessels

Potentization and storage vessels for all liquids were 500-ml narrow-necked bottles with standard ground joint (Schott Duran®, VWR International, Dietikon, Switzerland), with a conical shoulder, made from borosilicate glass with hydrolytic class 1, i.e., highly resistant against corrosion in neutral, basic, and acid environments. Bottles were closed with standard ground Duran® flat-head stoppers. All 80 vessels had been previously numbered permanently to enable retracing the use of every individual vessel during the entire study. All vessels had been previously tested for ion leaching by inductively coupled plasma mass spectrometry and found to be highly inert (contamination <100 ppb)[22]. Cleaning of the vessels and filling up of the samples were performed in a laboratory under a laminar flow box (BSB 4 A, Gelaire®, Flow Laboratories, Seven Hills NSW, Australia).

For the UV spectroscopy measurements, samples were filled into test tubes, i.e., Schott Fiolax®, 14-ml tubes, hydrolytic class 1, except for the first two measurements of $CuSO_4$, for which polyethylene test tubes were used. The test tubes were filled using pipettes (Falcon®, 10 ml, sterile, polystyrene pipettes) under the laminar flow box. Prior to the first use, all test tubes were cleaned three times with 18 MΩ water. The water remained in the test tubes for >1 week. The triple cleaning procedure was carried out to reduce possible ion leaching from the vessel wall.

### Production of Homeopathic Preparations and Controls

Homeopathic preparations and controls were produced using the multiple glass method and a laminar flow box according to the legal regulation for homeopathic remedies[23]. Potentization was performed by hand by horizontally shaking the vessel at a rate of about 2.7 Hz for 4 min before each dilution level.

Homeopathic preparations of $CuSO_4$ were produced in liquid phase as c preparations (i.e., centesimal, 100-fold dilution with each step) up to 30c. Homeopathic preparations of $S_8$ were produced in liquid phase as x potencies (i.e., decimal, 10-fold dilution), starting with $S_8$ 6x. Since $S_8$ is not soluble in water, 1x to 5x of $S_8$ were prepared as triturations, using lactose.

For both homeopathic preparations, $CuSO_4$ and $S_8$ independent water controls were produced by successing the potentization medium (solvent) at the same frequency and duration, but not diluting[24]. For $CuSO_4$ and $S_8$, 10 and 12 controls were used, respectively. To account for possible interferences during the production process, half of the controls were prepared before and half were prepared after the production of the homeopathic preparations. These controls account for all unspecific physicochemical effects, such as increased ion and air dissolution, air suspension, and radical formation, compared to unsuccussed solvent[24]. Specific effects of potentized solvent have been reported in biological models[25,26,27], indicating that by diluting and successing a solvent, it may become a homeopathic preparation itself. Therefore, we used only diluted, but not potentized, solvent as controls.

Randomization was effectuated by computer by randomly allocating the numbered potentization vessels to the dilution levels and the controls. Codes were only disclosed after data analysis was completed; thus, the measurements and data analysis were blinded. Additionally, homeopathic preparations and controls were externally indistinguishable.

### Interventions

For both $CuSO_4$ and $S_8$ homeopathic preparations and their controls, three separate sets of samples were filled into test tubes and exposed to three different types of interventions: (1) exposure to UV light of a sterilization lamp (Germicidal, HNS 30W OFR, Osram) for 12 h, (2) incubation at 37°C for 24 h, and (3) heating in a water bath at a temperature of 90°C for 15 min. All samples were allowed to cool down to room temperature prior to the measurement, i.e., 60 min for 37°C and 90 min for 90°C.

### UV-Spectroscopy Measurements

A Shimadzu UV PC 1601 spectrometer with a wavelength range from 190 to 1100 nm, equipped with an autosampler ASX-260, and a sipper was used. Prior to the measurements of the homeopathic preparations, comprehensive preparatory measurements were carried out to determine the influence of the following instrumental parameters on reproducibility: scan speed, wavelength of lamp change from visible (VIS) to UV lamp, instrumental drift, i.e., warm-up time, number of repetitions, i.e., with exchanged or same sample, and purge and sip time. Thus, instrumental tuning was optimized.

Light transmission of all samples was measured from 190 to 340 nm at medium scan speed. $CuSO_4$ from 6c to 30c and 10 controls, and $S_8$ from 10x to 30x and 12 controls, were measured.

All samples were measured once before the measurement was repeated. All samples were measured four times, except for the first two measurements of $CuSO_4$, which were repeated twice (three measurements). Before measuring the actual samples, five samples of distilled water (Aqua B. Braun®) were measured as run-in. The spectrometer was switched on 90 min prior to the measurement to allow a thorough warm-up and to minimize the instrument's drift. Before starting the measurements, a baseline calibration was carried out where the cuvette was filled with distilled water. The sip speed was set to "fast", sip time to 18 sec, and purge time to 20 sec. For purging, Aqua B. Braun® was used.

Six separate sets of samples of $CuSO_4$ and $S_8$ at each potentization level without exposure to external factors were measured at six different times: $CuSO_4$ was measured 4, 12, 19, 26, 33, and 110 days after production. $S_8$ was measured 5, 12, 19, 26, 91, and 256 days after production. The samples exposed to UV light and to incubation at 37°C were measured at room temperature 60 min after exposure and the samples heated to 90°C were allowed to cool down to room temperature for 90 min prior to the measurement. These measurements were conducted between 34 and 43 days after preparation for $CuSO_4$ (UV light 34 days, incubating to 37ºC 36 days, and heating to 90ºC 42 days), and between 21 and 25 days after preparation for $S_8$ (UV light 21 days, incubating to 37ºC 23 days, and heating to 90ºC 25 days).

### Data Analysis

The UV spectroscopy instrument was baseline calibrated with distilled water, i.e., the transmission of distilled water was set according to standard procedures to 100% for all wavelengths. The transmission values of the homeopathic preparations and controls were measured in relation to this baseline calibration. Since we used distilled water as a solvent for the homeopathic preparations and controls, all measured transmission values are slightly higher or lower than 100% throughout the whole spectrum.

Since we observed a slight instrumental drift during the first measurement of all samples, they were excluded from further analysis. All measurements were scanned visually for outliers, which were subsequently removed (0.62% of the data removed prior to uncoding). Such outliers may be due to, for example, bubbles in the cuvette. For each sample, the values were averaged across the two, respectively three, repeated measurements for each wavelength. A median, which is less sensitive to outliers than a mean, was calculated across two bands, i.e., from 190 to 340 nm and from 220 to 340 nm. The rationale for choosing two bands is that the instrumental noise is higher at the border of the measuring range of the instrument. Thus, values below 220 nm are less stable. On the other hand, the effect may be higher in this range. Therefore, we analyzed both measuring ranges.

Thus, for each measurement series (i.e., the values of one homeopathic preparation series and its controls measured on a specific day), the means and standard deviations (SD) were calculated for homeopathic preparations and controls and, subsequently, the resulting mean differences. The significance of this difference was tested by t-test, which does not assume equal variances. The Levene's test was calculated to compare variances between homeopathic preparations and controls. Additionally, each dilution level of a homeopathic preparation was tested against the controls. Due to multiple testing (25 tests for $CuSO_4$ and 21 tests for $S_8$), we only report significances at a reduced level of $p \leq 0.05/25 \leq 0.002$ according to Bonferroni.

Data of the measurements of unexposed samples were pooled as follows: first to third measurement series, fourth to sixth measurement series, as well as first to sixth measurement series. For this purpose, for each sample, the transmission value was averaged across the respective number of series and the same statistics as described above were calculated.

Tests for differences between unexposed and exposed homeopathic preparations were performed as follows. Since the baseline value of the instrument may vary between different measurement series, we calculated the mean value across the control samples as a measure of the instrumental factors and determined the difference between the mean value of the control samples of an exposed and unexposed series. This difference was used to correct for the difference between exposed and unexposed homeopathic preparations.

All statistics were calculated by the SPSS 15.0 software (SPSS Inc., Chicago).

### RESULTS

The results of the measurements and the statistics are shown in Tables 1–6 and Figs. 1 and 2.

For $CuSO_4$, we found several statistically significant mean differences, as well as variances between unexposed homeopathic preparations and controls at different points in time (Table 1). Significant differences always corresponded to a lower UV transmission and higher variance in homeopathic preparations than in controls. Pooling the data led to higher significances.

The absolute values may differ between measurements at different times due to the calibration of the UV spectroscopy instrument.

For $S_8$, no significant effects were found (Table 2). Compared to $CuSO_4$ preparations, $S_8$ samples had a higher variability in the UV transmission values.

Exposure of $CuSO_4$ homeopathic preparations to UV light or incubating at 37°C led to a significantly higher variance compared to controls (Table 3). In addition, the incubation at 37°C resulted in a significant difference in UV transmission and, again, lower values for homeopathic preparations (Table 3).

Exposure of $S_8$ homeopathic preparations to UV light or elevated temperatures did not lead to any significant effects (Table 4).

For each type of exposure, the UV light transmission of the homeopathic preparations of $CuSO_4$ was significantly further reduced compared to the unexposed homeopathic preparation of the closest age (Table 5). Incubating at 37ºC had the strongest effect. Even for $S_8$, a significant reduction in UV transmission was observed. It is also noteworthy that, in general, UV transmission was lower for exposed compared to unexposed homeopathic preparations of both substances. There were some discrete dilution levels that showed significantly reduced transmission values compared to the controls (Table 6).

### DISCUSSION

In our results, we found that UV transmission for $CuSO_4$ preparations that were exposed to UV light and elevated temperatures was partly significantly lower and more variable than in controls. For $S_8$ preparations, we did not observe any significant differences in transmission, except for the comparison between the measurements of the exposed and unexposed samples. To verify that the observed significant differences in UV light transmission were not generated by artifacts, the following aspects were considered.

#### Instrument

The UV spectrometer was a double-beam instrument that had an enhanced measurement stability compared to a single-beam instrument and, thus, an increased reproducibility. The reproducibility was optimized through comprehensive pilot studies, in which we determined the optimal measurement setup (scan speed, lamp change, sip speed, sip time, purge time, dwell time). After a warming-up period of 90 min and the first run of a measurement series, no instrumental drift was detectable, i.e., differences between runs were arbitrary.

Other factors like air humidity, room temperature, or the amount of dissolved oxygen in the preparations may influence the UV transmission in principle. However, these factors would have affected all preparations and controls in the same manner and can therefore be ruled out.

### TABLE 1
## Results of the Different Measurements of $CuSO_4$ Homeopathic Preparations and Controls without Exposure to External Factors*

| Measurement | Type | Wavelength 190–340 nm | | | | Wavelength 220–340 nm | | | |
|---|---|---|---|---|---|---|---|---|---|
| Age (days) | | Mean ± SD (%) | Mean Diff ± SEM (%) | *p* t-test | *p* Levene | Mean ± SD (%) | Mean Diff ± SEM (%) | *p* t-test | *p* Levene |
| 4 | C | 100.2514 ± 0.0445 | 0.0260 ± 0.0110 | 0.161 | 0.764 | 100.1411 ± 0.0200 | –0.0054 ± 0.0062 | 0.548 | 0.078 |
| | HP | 100.2254 ± 0.0550 | | | | 100.1465 ± 0.0312 | | | |
| 12 | C | 100.0421 ± 0.0428 | 0.0070 ± 0.0075 | 0.658 | 0.884 | 100.0391 ± 0.0381 | 0.0062 ± 0.0066 | 0.660 | 0.824 |
| | HP | 100.0351 ± 0.0373 | | | | 100.0329 ± 0.0329 | | | |
| 19 | C | 100.1954 ± 0.0229 | 0.0006 ± 0.0064 | 0.952 | 0.222 | 100.1767 ± 0.0221 | 0.0034 ± 0.0066 | 0.729 | 0.219 |
| | HP | 100.1948 ± 0.0321 | | | | 100.1733 ± 0.0331 | | | |
| 26 | C | 103.1653 ± 0.0183 | 0.0274 ± 0.0102 | **0.025** | 0.098 | 102.7035 ± 0.0256 | 0.0314 ± 0.0094 | **0.017** | 0.113 |
| | HP | 103.1378 ± 0.0509 | | | | 102.6721 ± 0.0471 | | | |
| 33 | C | 100.4460 ± 0.0360 | –0.0008 ± 0.0111 | 0.962 | 0.149 | 100.4327 ± 0.0341 | –0.0004 ± 0.0109 | 0.977 | 0.134 |
| | HP | 100.4468 ± 0.0554 | | | | 100.4331 ± 0.0546 | | | |
| 110 | C | 100.1226 ± 0.0815 | 0.0606 ± 0.0175 | 0.068 | 0.904 | 100.0756 ± 0.0641 | 0.0551 ± 0.0140 | **0.038** | 0.671 |
| | HP | 100.0621 ± 0.0873 | | | | 100.0205 ± 0.0702 | | | |
| Pooled 4–19 | C | 100.1629 ± 0.0305 | 0.0112 ± 0.0058 | 0.336 | 0.718 | 100.1189 ± 0.0202 | 0.0014 ± 0.0041 | 0.858 | 0.758 |
| | HP | 100.1518 ± 0.0290 | | | | 100.1175 ± 0.0203 | | | |
| Pooled 26–110 | C | 101.2446 ± 0.0290 | 0.0291 ± 0.0097 | **0.038** | **0.024** | 101.0706 ± 0.0205 | 0.0287 ± 0.0076 | **0.008** | **0.015** |
| | HP | 101.2156 ± 0.0487 | | | | 101.0419 ± 0.0381 | | | |
| Pooled all | C | 100.7038 ± 0.0245 | 0.0201 ± 0.0060 | 0.053 | 0.200 | 100.5948 ± 0.0154 | 0.0150 ± 0.0045 | **0.033** | 0.059 |
| | HP | 100.6837 ± 0.0301 | | | | 100.5797 ± 0.0225 | | | |

* Time is given in days after production of the homeopathic preparations (HP) and controls (C). Statistically significant values of the t-test or the Levene's test are displayed in bold typeface.

### TABLE 2
## Results of the Different Measurements of $S_8$ Homeopathic Preparations and Controls without Exposure to External Factors*

| Measurement | Type | Wavelength 190–340 nm | | | | Wavelength 220–340 nm | | | |
|---|---|---|---|---|---|---|---|---|---|
| Age (days) | | Mean ± SD (%) | Mean Diff ± SEM (%) | *p* t-test | *p* Levene | Mean ± SD (%) | Mean Diff ± SEM (%) | *p* t-test | *p* Levene |
| 5 | C | 100.3915 ± 0.0895 | 0.0118 ± 0.0187 | 0.716 | 0.933 | 100.3720 ± 0.0559 | 0.0169 ± 0.0145 | 0.444 | 0.681 |
| | HP | 100.3797 ± 0.0857 | | | | 100.3551 ± 0.0665 | | | |
| 12 | C | 100.1814 ± 0.1013 | –0.0224 ± 0.0198 | 0.534 | 0.564 | 100.2039 ± 0.0820 | –0.0193 ± 0.0165 | 0.511 | 0.873 |
| | HP | 100.2038 ± 0.0908 | | | | 100.2232 ± 0.0757 | | | |
| 19 | C | 100.0973 ± 0.1259 | –0.0120 ± 0.0209 | 0.777 | 0.310 | 100.0894 ± 0.1232 | –0.0161 ± 0.0192 | 0.695 | 0.234 |
| | HP | 100.1093 ± 0.0956 | | | | 100.1056 ± 0.0882 | | | |
| 26 | C | 99.6260 ± 0.1416 | –0.0151 ± 0.0217 | 0.748 | 0.510 | 99.6274 ± 0.1391 | –0.0198 ± 0.0215 | 0.670 | 0.442 |
| | HP | 99.6411 ± 0.0995 | | | | 99.6471 ± 0.0985 | | | |
| 91 | C | 100.1295 ± 0.1643 | –0.0128 ± 0.0405 | 0.840 | 0.714 | 100.0407 ± 0.1601 | –0.0167 ± 0.0403 | 0.787 | 0.785 |
| | HP | 100.1422 ± 0.1855 | | | | 100.0574 ± 0.1846 | | | |
| 256 | C | 99.8571 ± 0.1649 | –0.0827 ± 0.0341 | 0.172 | 0.743 | 99.8454 ± 0.1665 | –0.0854 ± 0.0338 | 0.161 | 0.770 |
| | HP | 99.9398 ± 0.1561 | | | | 99.9308 ± 0.1550 | | | |
| Pooled 5–19 | C | 100.2234 ± 0.0675 | –0.0075 ± 0.0148 | 0.761 | 0.818 | 100.2218 ± 0.0664 | –0.0062 ± 0.0128 | 0.792 | 0.535 |
| | HP | 100.2310 ± 0.0680 | | | | 100.2280 ± 0.0587 | | | |
| Pooled 26–256 | C | 99.8709 ± 0.1443 | –0.0368 ± 0.0292 | 0.477 | 0.948 | 99.8378 ± 0.1431 | –0.0406 ± 0.0293 | 0.431 | 0.992 |
| | HP | 99.9077 ± 0.1338 | | | | 99.8784 ± 0.1343 | | | |
| Pooled all | C | 100.0471 ± 0.0928 | –0.0222 ± 0.0172 | 0.494 | 0.712 | 100.0298 ± 0.0921 | –0.0234 ± 0.0176 | 0.472 | 0.711 |
| | HP | 100.0693 ± 0.0787 | | | | 100.0532 ± 0.0808 | | | |

* Time is given in days after production of the homeopathic preparations (HP) and controls (C). No statistical significances were found.

### TABLE 3
## Results of the Different Measurements of $CuSO_4$ Homeopathic Preparations and Controls that were Exposed to External Factors*

| Measurement | Type | Wavelength 190–340 nm | | | | Wavelength 220–340 nm | | | |
|---|---|---|---|---|---|---|---|---|---|
| | | Mean ± SD (%) | Mean Diff ± SEM (%) | *p* t-test | *p* Levene | Mean ± SD (%) | Mean Diff ± SEM (%) | *p* t-test | *p* Levene |
| UV light | C | 100.2117 ± 0.0370 | 0.0253 ± 0.0156 | 0.205 | **0.010** | 100.1900 ± 0.0352 | 0.0270 ± 0.0149 | 0.156 | **0.006** |
| | HP | 100.1864 ± 0.0781 | | | | 100.1630 ± 0.0745 | | | |
| 37° Incubation | C | 100.3068 ± 0.0377 | 0.0636 ± 0.0201 | **0.010** | **0.005** | 100.2771 ± 0.0389 | 0.0512 ± 0.0190 | **0.030** | **0.013** |
| | HP | 100.2432 ± 0.1004 | | | | 100.2259 ± 0.0948 | | | |
| 90° 15 min | C | 100.4706 ± 0.1054 | 0.0545 ± 0.0210 | 0.185 | 0.481 | 100.3876 ± 0.0757 | 0.0302 ± 0.0181 | 0.327 | 0.174 |
| | HP | 100.4162 ± 0.1048 | | | | 100.3574 ± 0.0904 | | | |

* UV light for 24 h, incubation at 37ºC for 24 h, and heating to 90ºC for 15 min. There were significant differences between homeopathic preparations of $CuSO_4$ and controls after heating to 37ºC and exposing to UV light. Significant results are displayed in bold typeface.

### TABLE 4
## Results of the Different Measurements of $S_8$ Homeopathic Preparations and Controls that were Exposed to External Factors*

| Measurement | Type | Wavelength 190–340 nm | | | | Wavelength 220–340 nm | | | |
|---|---|---|---|---|---|---|---|---|---|
| | | Mean ± SD (%) | Mean Diff ± SEM (%) | *p* t-test | *p* Levene | Mean ± SD (%) | Mean Diff ± SEM (%) | *p* t-test | *p* Levene |
| UV light | C | 100.3285 ± 0.0725 | 0.0043 ± 0.0159 | 0.872 | 0.843 | 100.3100 ± 0.0683 | –0.0014 ± 0.0131 | 0.952 | 0.864 |
| | HP | 100.3242 ± 0.0728 | | | | 100.3115 ± 0.0601 | | | |
| 37° Incubation | C | 100.0278 ± 0.0673 | 0.0303 ± 0.0103 | 0.186 | 0.060 | 100.0183 ± 0.0639 | 0.0247 ± 0.0101 | 0.255 | 0.106 |
| | HP | 99.9975 ± 0.0470 | | | | 99.9936 ± 0.0462 | | | |
| 90° 15 min | C | 100.1676 ± 0.1238 | 0.0205 ± 0.0196 | 0.621 | 0.472 | 100.1238 ± 0.1208 | 0.0002 ± 0.0176 | 0.996 | 0.307 |
| | HP | 100.1470 ± 0.0899 | | | | 100.1236 ± 0.0808 | | | |

* UV light heating to 37 and 90ºC. No statistical significances were found.

### TABLE 5
## Statistical Comparison between Unexposed and Exposed Homeopathic Preparations (without Controls)*

| Substance | Exposure | Wavelength 190–340 nm | | Wavelength 220–340 nm | |
|---|---|---|---|---|---|
| | | Mean Diff ± SEM (%) | *p* t-test | Mean Diff ± SEM (%) | *p* t-test |
| $CuSO_4$ | UV | –0.0260 ± 0.0125 | **0.049** | –0.0275 ± 0.0128 | **0.043** |
| | 37° | –0.0643 ± 0.0198 | **0.003** | –0.0517 ± 0.0177 | **0.008** |
| | 90° | –0.0552 ± 0.0188 | **0.007** | –0.0306 ± 0.0155 | 0.061 |
| Sulfur | UV | –0.0194 ± 0.0227 | 0.402 | –0.0183 ± 0.0193 | 0.355 |
| | 37° | –0.0454 ± 0.0197 | **0.032** | –0.0445 ± 0.0195 | **0.034** |
| | 90° | –0.0356 ± 0.0177 | 0.058 | –0.0200 ± 0.0148 | 0.194 |

* There were three types of exposure: UV, irradiation to UV light; 37º, incubation at 37ºC; and 90º, heating to 90ºC. The reference measurement without exposure is the one closest in time. For $CuSO_4$, this was the measurement at day 33 after production and for sulfur, the one at day 26. Significant results are displayed in bold. Data were normalized to the corresponding control samples.

**TABLE 6**
**Significant Individual Potency Levels of $CuSO_4$ and $S_8$ Compared to the Controls by a t-Test**

| Substance | Age (Days) or Intervention | 190–340 nm | 220–340 nm |
|---|---|---|---|
| $CuSO_4$ | 26 | C9***, C17*, C24*, C29 | C9, C17, C24 |
| | 33 | | C17 |
| | 110 | | C18 |
| | UV | C24 | C24 |
| | 37° | C22*, C24 | C22*, C24 |
| Sulfur | 5° | | D10 |

Note: Due to multiple testing, a reduced *p* level of ≤0.002 (corresponding to a Bonferroni corrected *p* level of 0.05) had to be fulfilled to establish statistical significance (* means $p \leq 0.0001$, *** means $p \leq 0.000001$). Only measurement series with significant dilution levels are displayed.

### Contamination

We used vessels of hydrolytic class 1 (Schott Duran® vessels, Fiolax® test tubes), which are highly resistant to leaching. Moreover, the homeopathic preparations as well as the controls were prepared in vessels (Schott Duran®) that had previously been tested for ion leaching by inductively coupled, plasma mass spectrometry and it was shown that contamination was negligible (<100 ppb). Therefore, contamination is not an explanation for the observed effects. Additionally, prior to their use, all vessels were cleaned with autoclaved, deionized, and distilled water. Even if leaching had taken place, due to the randomization of the vessels, it would have affected homeopathic preparations and controls similarly and can therefore be excluded.

### Experimenter's Influence

All samples were blinded and blinding was only disclosed after data analysis was completed. Moreover, all measurements were carried out in a random order.

### Variability Depending on the Course of Time and Type of Homeopathic Preparation

The differences between homeopathic preparations and controls vary between measurements. It seems that for older homeopathic preparations, the differences are more significant. It is difficult to explain this variation, which may be a specific property of the homeopathic preparations. We think that it is unlikely that this is due to artifacts in the measurement, because all data were thoroughly screened for such artifacts before unblinding.

It is also difficult to explain why $S_8$ does not display the same behavior. This may be due to the different dilution level or due to the different primary substance.

It is, however, the third time that $CuSO_4$ homeopathic preparations were significantly different compared to controls and transmission was always lower for homeopathic preparations. Thus, evidence is accumulating that this is a real effect.

In summary, trivial artifacts such as air humidity, dissolved oxygen, contamination, and experimenter's influence are not causal for the differences between homeopathic preparations and controls. If the observed effects are not correlated to artifacts, how can they be interpreted?

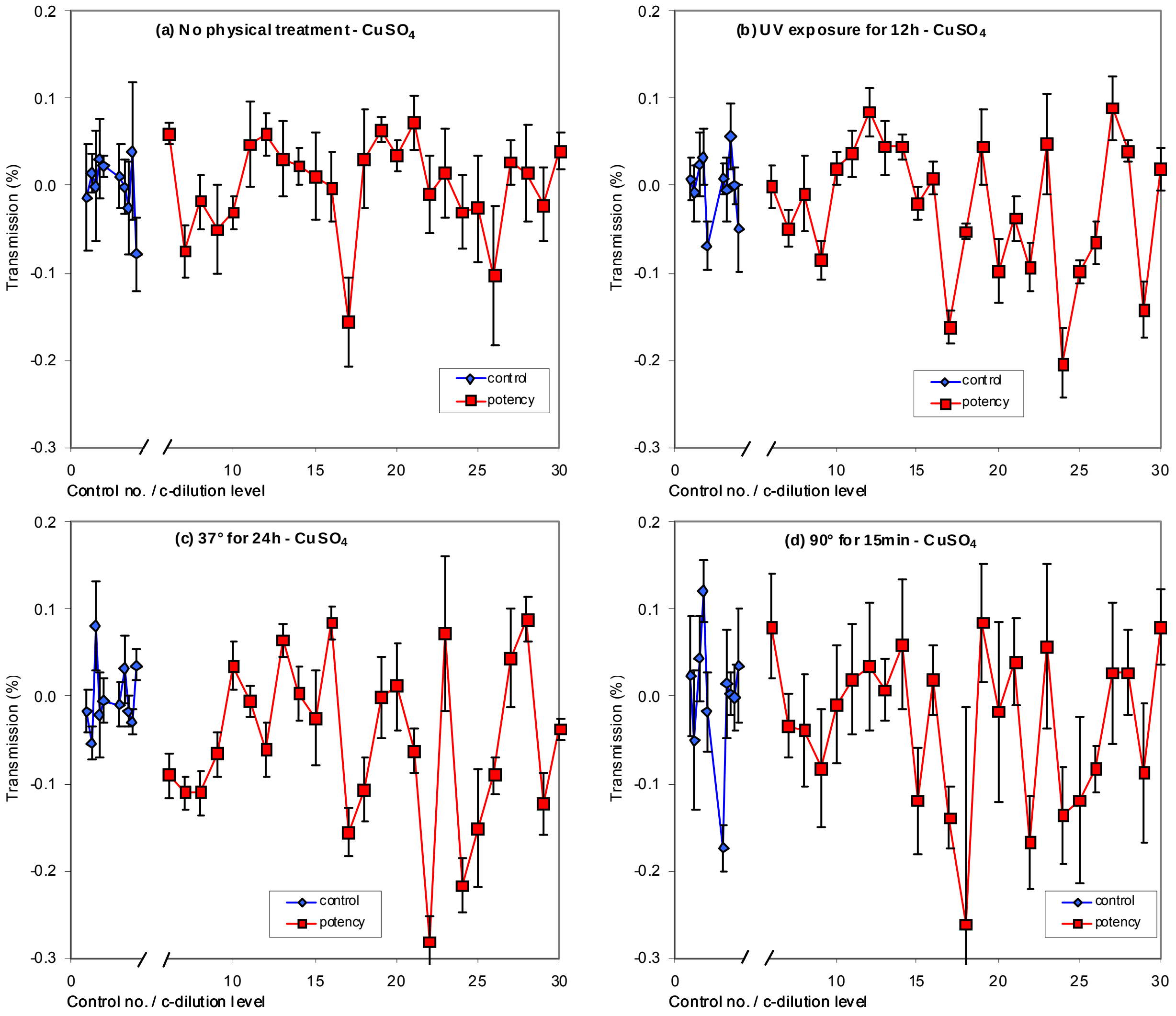

**FIGURE 1.** $CuSO_4$ homeopathic preparations and controls: (a) without exposure to physical treatment (day 33, ns), (b) exposure to 12 h UV light (Levene *p* = 0.006, (c) 24 h incubation at 37ºC (t-test *p* = 0.03, Levene *p* = 0.013), and (d) after 15 min heating to 90ºC in a water bath (ns). The median values of UV transmissions from 220 to 340nm are displayed in %. For better comparability, the mean transmission of the controls was subtracted from all values. The x-axis shows the potentization level of the homeopathic preparations. The controls are on the left in the order they were produced, the homeopathic preparations on the right side. The whiskers correspond to the standard deviation.

### Dynamization Hypothesis

Homeopathic preparations that had been exposed to external factors had lower UV transmission values compared to controls. This signifies that a higher amount of light was absorbed by the homeopathic preparations. In general, absorption is understood as either an electron being lifted to a higher energy level by a quantum of light or an increase in the vibrational energy status of a molecule. This may lead to a less structured or more dynamic molecular state of the sample. In the case of UV absorption in water, the absorption edge between 160 and 200 nm corresponds to an electronic transition between nonbonding and antibonding states ($n \rightarrow \sigma^*$) of electrons located in the lone pairs on the oxygen atom in the water molecule[28]. The nonbonding electrons involved in this transition are the same electrons that act as hydrogen acceptors during formation of intermolecular hydrogen bonds. Thus, the absorption also depends on the structure of water; higher temperatures (implying weaker H-bonds) lead to increased UV absorption[29].

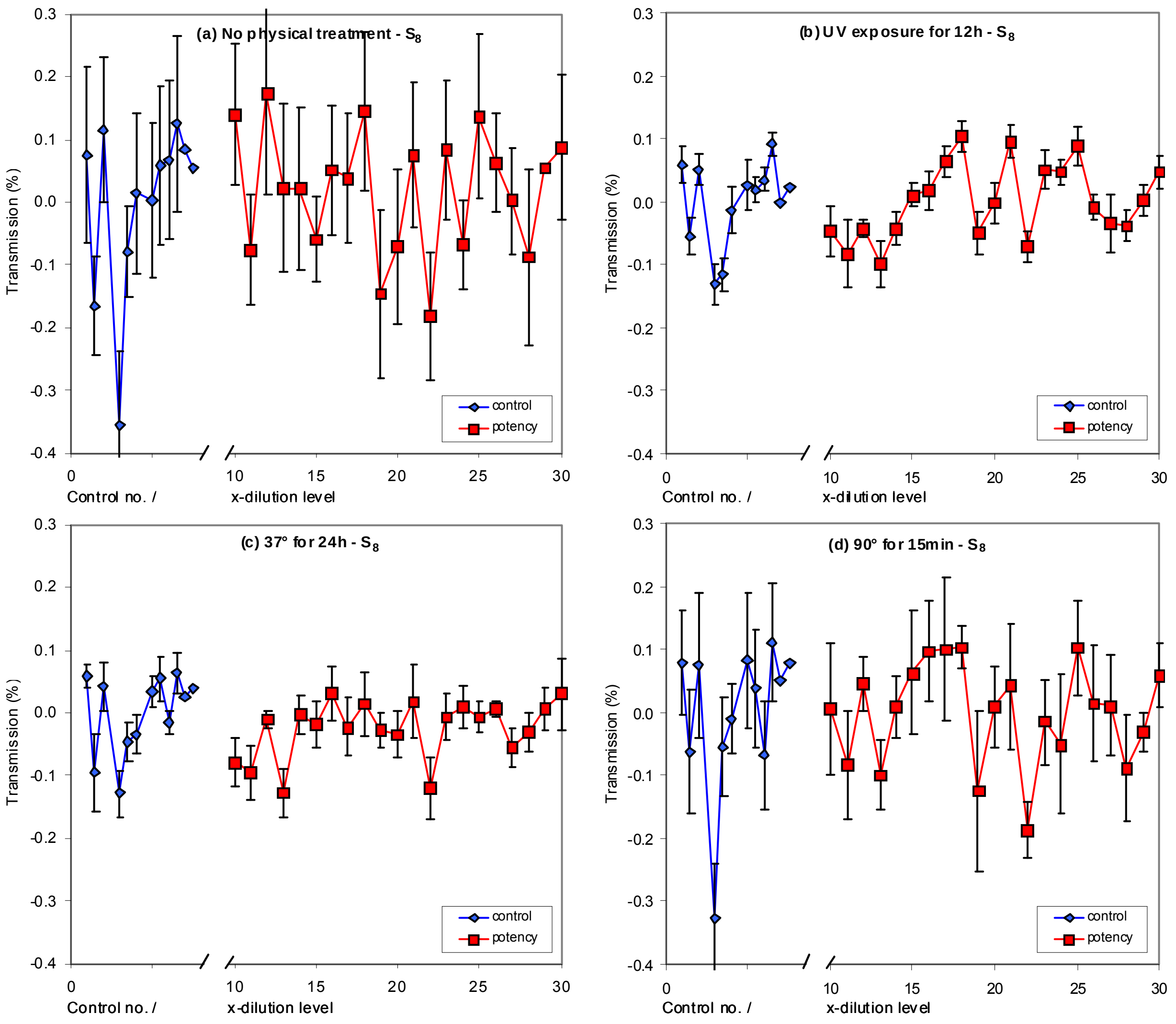

**FIGURE 2.** $S_8$ homeopathic preparations and controls: (a) without exposure to physical treatment (day 26, ns), (b) after exposure to 12 h UV light (ns), (c) after 24 h incubation at 37ºC (ns), and (d) after 15 min heating to 90ºC in a water bath (ns).

When considering the results for homeopathic preparations that were exposed to external factors, especially incubating at 37ºC, it may be hypothesized that homeopathic preparations retain a higher intermolecular energy level. Although heating itself leads to an increased intermolecular dynamics, the latter should return to the initial state once the temperature returns to baseline. If after exposure to external factors homeopathic preparations have a higher absorption than the controls and also compared to the unexposed homeopathic preparation, these effects may be interpreted as a higher intermolecular energy level or dynamics that remains even after exposure. Since the exposures were applied to homeopathic preparations and controls at the same time, the origin for the difference needs to be sought in the homeopathic preparations.

Additionally, it seems that the effect depends on the type of the exposure. We found that incubating at 37ºC had the strongest effect. In contrast, heating at 90ºC had the weakest effect.

There is a difference in the effects between the $CuSO_4$ and the $S_8$ homeopathic preparations. The reason for this is yet unclear. $S_8$ preparations and controls (Table 2) showed a much higher variability than $CuSO_4$ preparations (Table 1). This higher variability makes it statistically more difficult to detect the small differences between homeopathic potencies and controls, which might be a reason for the difference. Another explanation may be the different dilution factor; centesimal for $CuSO_4$ and decimal for $S_8$ preparations.

### Other Investigations of Homeopathic Preparations with UV Spectroscopy

To the best of our knowledge, this is the first investigation with UV spectroscopy where homeopathic preparations were subjected to external physical factors. We did not find studies in the literature to which our results for the exposed samples could be compared.

Several studies observed differences between homeopathic preparations and controls with UV spectroscopy[16,17,18,19,20,21]. In general, higher UV light absorption for homeopathic preparations was reported; these data are therefore in agreement with our results.

### Variability

Our results showed a higher variability in transmission values for homeopathic $CuSO_4$ preparations compared to the controls. External factors such as irradiation with UV light and incubation at 37°C increased this difference in variability. This may be indicative that some discrete dilution levels might have specific properties as described by Kolisko[30] and others[27,31,32]. These studies identified a discontinuous pattern with peaks and troughs, depending on the potentization level. This could explain the higher variability of the homeopathic $CuSO_4$ preparations compared to the controls after exposing homeopathic preparations and controls to external factors.

### Time

Homeopathic preparations or diluent may undergo some modifications during the course of time. In our study, the first three measurements of homeopathic preparations and controls of $CuSO_4$ showed no significant differences, while the pooled data of the last three measurements did. Maybe some time is required for an effect of the homeopathic preparations to develop. This effect was also previously observed in another study[33].

## CONCLUSION

After some time of storage, unexposed homeopathic preparations of $CuSO_4$ showed significantly lower UV transmission values and higher variance than corresponding controls. Incubation at 37°C increased this effect, while exposure to UV light increased the variance in homeopathic preparations.

The UV light transmission of the exposed homeopathic preparations of $CuSO_4$ compared to the unexposed homeopathic preparations was reduced for each form of exposure. Incubating at 37°C had the strongest effect and was even significant for $S_8$, which otherwise did not show significant effects.

The findings of this study show that exposure to external factors may affect homeopathic preparations.

The lower transmission values may indicate that the diluent is less structured or more dynamic after homeopathic potentization and that higher intermolecular energy is retained after exposure to physical factors.

Further research on this topic is highly desirable.

### ACKNOWLEDGMENT

This study was partly supported by the Asta Blumfeldt Foundation, Arlesheim, Switzerland. The sponsor had no influence whatsoever upon design, conduct, evaluation, and manuscript of this investigation.

We like to thank Agnès Deglon for providing language assistance.
